# An Ultrasensitive Ethanol Gas Sensor Based on a Dual-Nanoparticle In_2_O_3_/SnO_2_ Composite

**DOI:** 10.3390/s24237823

**Published:** 2024-12-07

**Authors:** Cheng Zhang, Ze Zhang, Yao Tian, Lingmin Yu, Hairong Wang

**Affiliations:** 1 State Key Laboratory for Manufacturing Systems Engineering, Xi’an 710049, China; eeessseee@stu.xjtu.edu.cn (C.Z.); zhangze0722@126.com (Z.Z.); 17736301096@163.com (Y.T.); 2 School of Mechanical Engineering, Xi’an Jiaotong University, Xi’an 710049, China; 3 School of Materials and Chemical Engineering, Xi’an Technological University, Xi’an 710021, China; ylmyl@163.com

**Keywords:** nanocomposite, n-n heterojunction, gas sensors, ethanol, low detection limit

## Abstract

As a VOC, ethanol can be found in human exhaled breath, and its concentration can be used as a biomarker of human liver disease. To detect trace-level concentrations of ethanol, an ultrasensitive ethanol sensor was developed based on a dual-nanoparticle In_2_O_3_/SnO_2_ composite that was prepared by hydrothermal synthesis, and its suspension was dipped on a flat electrode to form a gas sensor. The nanocomposite was characterized by an SEM (scanning electron microscope), XRD (X-ray diffraction), and a TEM (transmission electron microscope), and the nanoparticle structure was observed. The experimental results showed that gas sensors based on the In_2_O_3_/SnO_2_ nanocomposite had higher responses compared to sensors based on pure In_2_O_3_. Among the nanocomposites, the one with a In_2_O_3_-to-SnO_2_ mol ratio of 1:8 was used in the sensor with the highest response of 1.41 to 100 ppb ethanol at 150 °C, which also exhibited good repeatability. The ultrasensitive response to ethanol can be attributed to the faster electron migration rate and the increase in oxygen-absorbing sites caused by the n-n heterojunction in the nanocomposite. Due to its low detection limit, good repeatability, and relatively high responses in high humidity, this sensor has a potential application in exhaled breath detection.

## 1. Introduction

Ethanol is a VOC (volatile organic compound), and it is widely used in many fields such as chemical industries, pharmacology, motor vehicle fuel, food industries, liquors, beverages, and so on [1]. Ethanol gas is also present in breath exhaled from the human body [2]. Like many other kinds of VOCs in human exhaled gases [3,4,5,6], the concentration of ethanol may correspond to the health of the human body [7,8,9,10]. For example, ethanol gas has been used as a biomarker of liver disease [11]. To realize disease screening by exhaled breath analysis, one precondition is to detect the concentration of ethanol gas fast and accurately. Turner C et al. used SIFT-MS (selected-ion flow-tube mass spectrometry) to monitor the volatile compounds in the exhaled breath of 30 volunteers (19 males and 11 females) over a 6-month period and found that the mean ethanol level for all samples was 196 ppb (parts per billion) with a standard deviation of 244 ppb, and the ethanol concentrations of all breath samples ranged from 0 to 1663 ppb [12]. To detect the trace-level ethanol that exists in complex exhaled gases, a sensor must have a very low detection limit, high selectivity, and good anti-humidity ability. So far, it is still quite difficult to find a gas sensor with all these merits.

Many studies have shown that MOS (metal oxide semiconductor) sensors are effective for ethanol gas detection [13]. In 1962, Seiyama et al. developed the first metal oxide-based gas sensor [14]. Nowadays, metal oxide sensors have been thoroughly studied and widely applied [15,16,17,18,19,20,21]. Frequently used metal oxide materials include SnO_2_ [22,23], In_2_O_3_ [24], ZnO [25], TiO_2_, and so on [26,27,28,29,30,31,32]. In_2_O_3_ is a typical n-type material with a wide band gap and low resistivity. P. Van Tong et al. synthesized porous In_2_O_3_ nanorods and developed a gas sensor based on this nanomaterial, which has excellent CO-sensing performance [33]. Furthermore, In_2_O_3_ is also used to detect gas at ultralow concentrations. A gas sensor based on the hierarchical branch-like In_2_O_3_ nanomaterial has obvious responses to ppb-level ozone [34]. Different microstructures can also be beneficial for gas responses. A larger specific surface area can adsorb more gas molecules, resulting in higher response values and lower detection limits. W. Zhu et al. found that In_2_O_3_ nanospheres have high responses, long-term stability, a short response time for ethanol, and good selectivity compared to other materials [35]. In order to obtain better response characteristics for ethanol, researchers turned to heterojunctions by combining In_2_O_3_ with other metal oxides; e.g., O. K. Zhang et al. prepared hierarchical ZnO-In_2_O_3_ heterojunctions, and gas sensors based on these heterojunctions had high sensitivity and selectivity to ethanol [36]. The work functions of the two metal oxide materials are different, and the energy band bends at contact. Electron mobility increases, so more oxygen vacancies are generated when adsorbing air, more free electrons are released when contacting the target gas, and better response characteristics are obtained. Due to its wide band gap and stable chemical properties, SnO_2_ is widely used in VOC gas sensors [37]. The responses of gas sensors based on In_2_O_3_-SnO_2_ nanocomposites are higher than those of pure SnO_2_ for detecting formaldehyde, and their improved sensing properties were actually attributed to the n-n heterojunction and the synergistic interactions between In_2_O_3_ and SnO_2_ [38]. D. An et al. prepared a series of materials. In_2_O_3_-SnO_2_ nanocomposites were synthesized by the hydrothermal method. A sensor based on an In_2_O_3_–SnO_2_ composite exhibited high responses to n-butanol gas. [39]. Y. Liu et al. prepared In_2_O_3_-SnO_2_ by the hydrothermal method, and the test results showed that a sensor made of this material had a good response performance for ethanol gas [40]. Illuminated by these findings, in this study, we developed an ultrasensitive ethanol sensor based on a nanocomposite formed by the nanoparticle metal oxides In_2_O_3_ and SnO_2_, which were prepared via hydrothermal synthesis, and attempted to detect ethanol at the hundred ppb level in a humid environment like exhaled breath.

## 2. Experimental Section

### 2.1. Nanocomposite Preparation

We prepared the dual-nanoparticle In_2_O_3_/SnO_2_ composite by the hydrothermal method as follows [41]. First, 100 mL of distilled water was placed in a beaker. Then, solutions of 10 mmol NaCO_3_ and 5 mmol SnCl_4_·5H_2_O were put into the beaker. It was stirred in a magnetic mixer for 2 h. After that, the mixed solution in the vessel was poured into a PTFE (polytetrafluoroethylene)-lined reactor. Then, the reactor was placed in a drying oven and heated at a constant temperature of 160 °C for 12 h [39]. After naturally cooling to room temperature, a suspension was obtained, and it was processed by washing in anhydrous ethanol and deionized water several times. The resultant was ultrasonically cleaned for 10 min and was then centrifuged at 4000× *g* rpm (revolutions per minute) for 30 min. The above cleaning process was repeated a couple of times. The remaining suspension was placed in a drying oven to thermostatically dry at 80 °C for 12 h until the suspension completely became a white powder. Finally, to calcinate the precipitate, the heating rate was set as 2 °C min^−1^. The temperature was increased to 400 °C and maintained for 2 h. The result was a pure SnO_2_ nanostructure, which was marked as 1#.

Then, solutions of 10 mmol NaCO_3_ and 5 mmol In(NO_3_)_3_ were put into a vessel with 100 mL of distilled water, and they were stirred vigorously in a magnetic mixer for 2 h. The mixed solution was put into the PTFE-lined reactor, and the reactor was heated at a constant temperature of 160 °C for 12 h in a drying oven [42]. Using the same process as before, after naturally cooling to room temperature, a suspension was obtained, and it was processed by washing with anhydrous ethanol and deionized water several times. The resultant was cleaned via centrifugation for 10 min. The above cleaning step was repeated a couple of times. The remaining suspension was placed in a drying oven to thermostatically dry at 80 °C for 12 h until the suspension completely became a white powder. Finally, to calcinate the precipitate, the heating rate was set as 2 °C min^−1^. The temperature was increased to 400 °C and maintained for 2 h. The result was a pure In_2_O_3_ nanostructure, which was marked as 6#.

**Figure 1:**
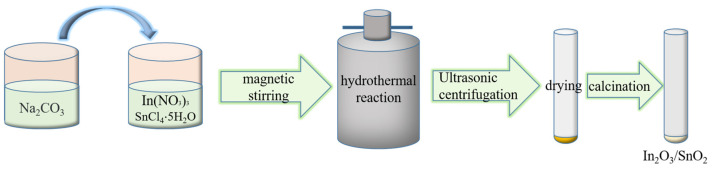
A schematic of the nanocomposite preparation process.

To prepare the In_2_O_3_/SnO_2_ nanocomposites, four groups of solutions, which were 1 mmol SnCl_4_·5H_2_O and 4 mmol In(NO_3_)_3_; 2 mmol SnCl_4_·5H_2_O and 3 mmol In(NO_3_)_3_; 3 mmol SnCl_4_·5H_2_O and 2 mmol In(NO_3_)_3_; and 4 mmol SnCl_4_·5H_2_O, 1 mmol In(NO_3_)_3_, and 10 mmol NaCO_3_, were selected and poured into vessels with 100 mL of distilled water. With the same processes used for samples 1# and 6#, four kinds of In_2_O_3_/SnO_2_ nanocomposites with different mol ratios were prepared and marked as 2#, 3#, 4#, and 5#, as shown in Table 1. The nanocomposite preparation process is shown schematically in Figure 1.

### 2.2. Preparation of the Gas Sensors and Test Methods

**Figure 2:**
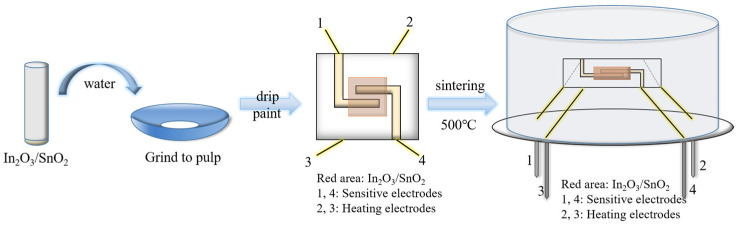
The process of preparing the gas sensors on the flat ceramic sheet.

The powders of the above sensitive nanomaterials were put into test tubes, and a moderate amount of deionized water was added to prepare suspensions. A planar ceramic sheet measuring 3 × 3 × 0.25 mm had two sensitive electrodes on one side and heating electrodes on another side. A suspension of sensitive material was manually coated on the sensitive electrodes by drop coating. The sensitive-material coating was heated in an oven at 500 °C for 2 h. Finally, the four pins of the ceramic sheet were welded to the sensor TO packaging with soldering tin. The preparation process is shown in Figure 2.

**Figure 3:**
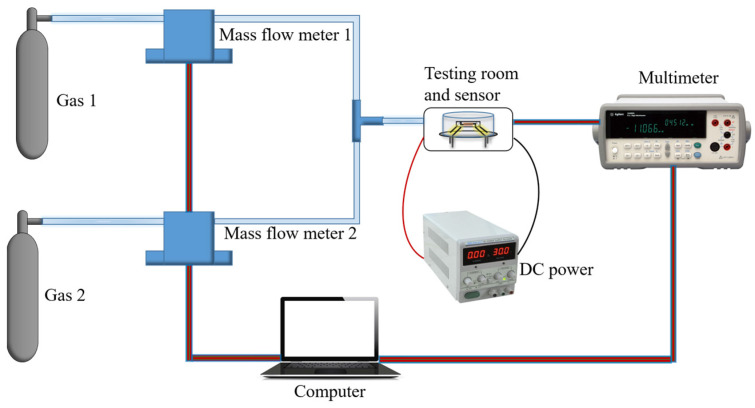
The real-time dynamic valve system.

The sensors were tested using a gas distribution system, as shown in Figure 3. Two mass flow meters (Sevenstar CS200A) controlled by a computer provided different flow rates for ethanol and dry air. The mixed gas flowed into the cylindrical gas chamber in which the gas sensor was installed at 200 sccm (standard cubic centimeters per minute). A DC power supply was used to apply a voltage to the two heating electrodes, providing working temperatures for the gas sensor. The resistance between the two sensitive electrodes of the gas sensor was monitored by a digital multimeter (Agilent 34970 A). For the gas sensor, the resistance between the two sensitive electrodes in pure air was *R_a_*, the resistance in an atmosphere with the target gas was *R_g_*, and the response of the sensor based on an n-type metal oxide was defined using Equation (1).

(1) $$Response=R_{a}/R_{g}$$

In the test, 2 ppm ethanol in dry air was marked as gas 1 and dry air was marked as gas 2. The ethanol concentration was changed by controlling the flow rate ratio of the two mass flow meters; e.g., an ethanol concentration of 100 ppb could be achieved with 5% gas 1 (2 ppm ethanol) and 95% gas 2 (dry air).

## 3. Results and Discussion

### 3.1. Characterization

**Figure 4:**
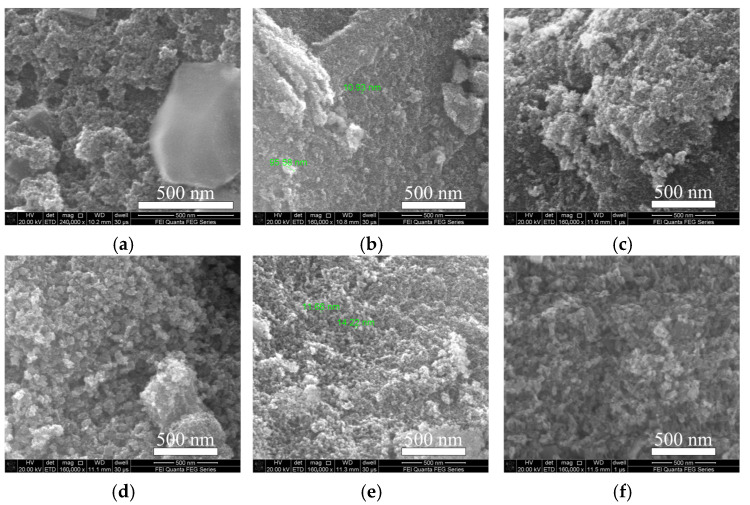
SEM images of (**a**) pure SnO_2_; (**b**–**e**) In_2_O_3_/SnO_2_ nanocomposites with ratios of 1:8, 2:6, 3:4, and 2:1; and (**f**) pure In_2_O_3_.

The prepared materials were then characterized and analyzed in different ways. FESEM (field emission scanning electron microscopy) images were obtained on a Quanta 250FEG microscope with an operating voltage of 20 kV. As shown in Figure 4, the In_2_O_3_, SnO_2_, and In_2_O_3_/SnO_2_ composites were irregular nanoparticles with diameters of about 10 nm. The same 500 nm scale bar is used for all images. Some of the particles were clustered together, most of them showed bumpy surfaces, and the sizes of several of the more obvious nanoparticles were measured (about ten nm to tens of nm).

**Figure 5:**
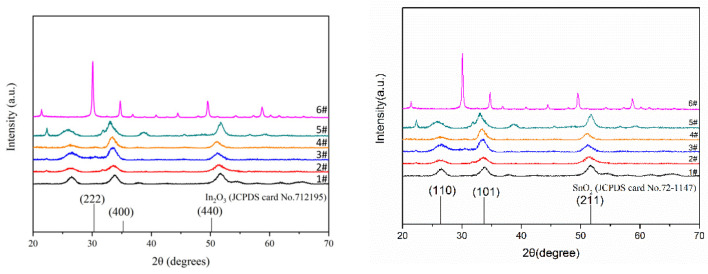
XRD patterns of In_2_O_3_, SnO_2_, and nanocomposites of In_2_O_3_/SnO_2_ with different ratios.

The XRD (X-ray diffraction) patterns of the In_2_O_3_, SnO_2_, and In_2_O_3_/SnO_2_ composites are shown in Figure 5. They were obtained using a Bruker D8 ADVANC instrument (Cu Kα, 2.2 kW, λ = 1.54056 Å) at 2θ from 20° to 70°. The angles of the main characteristic peaks were observed at 2θ values of 30.63°, 35.54°, and 51.04°, which corresponded to the (222), (400), and (440) planes, respectively, when compared with an In_2_O_3_ standard card (JCPDS71-2195). The material belonged to a cubic crystal-type iron manganese ore type with an average grain size of 29 nm. The SnO_2_ pattern was compared with the standard card JCPDS72-1147, and the angles of the main characteristic peaks were observed at 2θ values of 26.57°, 33.92°, and 51.74°, which corresponded to the (110), (101), and (211) planes. The crystal type was a tetragonal rutile type, and the average grain size was 6 nm. Figure 5 shows that the peak was obvious during the detection of pure In_2_O_3_. When SnO_2_ was present, the corresponding peak was covered, probably because of the small content of In_2_O_3_. Finally, the curve of the composite material only had a relatively obvious peak of SnO_2_. It is also possible that the In_2_O_3_ in the composite material existed in the interior of the SnO_2_, making the curve of the SnO_2_ more obvious.

### 3.2. Gas-Sensing Characteristics

**Figure 6:**
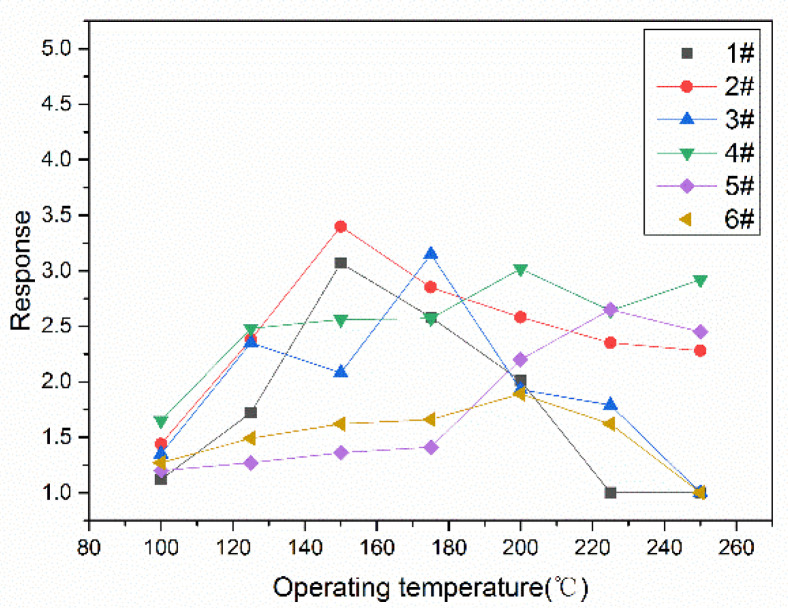
The optimal operating temperatures of different sensors for 1 ppm ethanol.

Next, a series of gas-sensitive performance tests were performed on the sensors. Figure 6 describes the relations between the responses and the operating temperatures of the In_2_O_3_/SnO_2_ sensors for 1 ppm ethanol at operating temperatures ranging from 100 °C to 250 °C. The resistances of all sensors changed at different operating temperature values, and the reason for the resistance changes induced by the changes in the sensor materials at the same operating temperatures was the n-n heterojunction. The responses of the sensors reached their maximum values at their optimal operating temperatures. The optimal operating temperature of In_2_O_3_/SnO_2_ sensor sample 2# was 150 °C, as shown in Figure 6.

**Figure 7:**
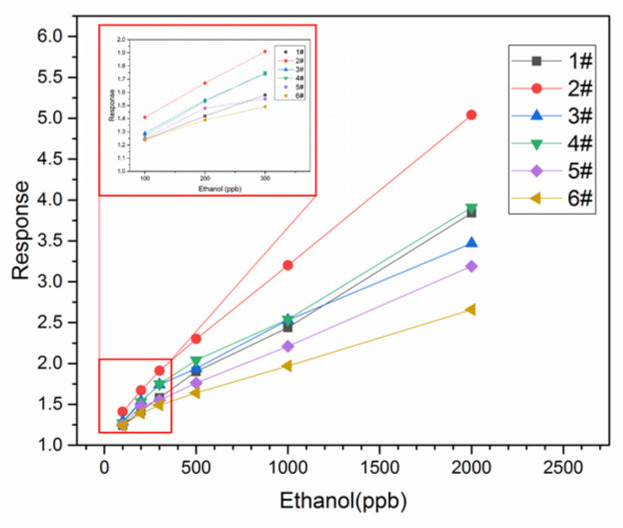
The responses to different concentrations of ethanol.

The responses of the sensors to ethanol from 100 ppb to 2000 ppb were tested at the optimal operating temperature, as shown in Figure 7. The responses of all sensors increased with increasing concentrations, and sample 2# had the highest response of all sensors.

**Figure 8:**
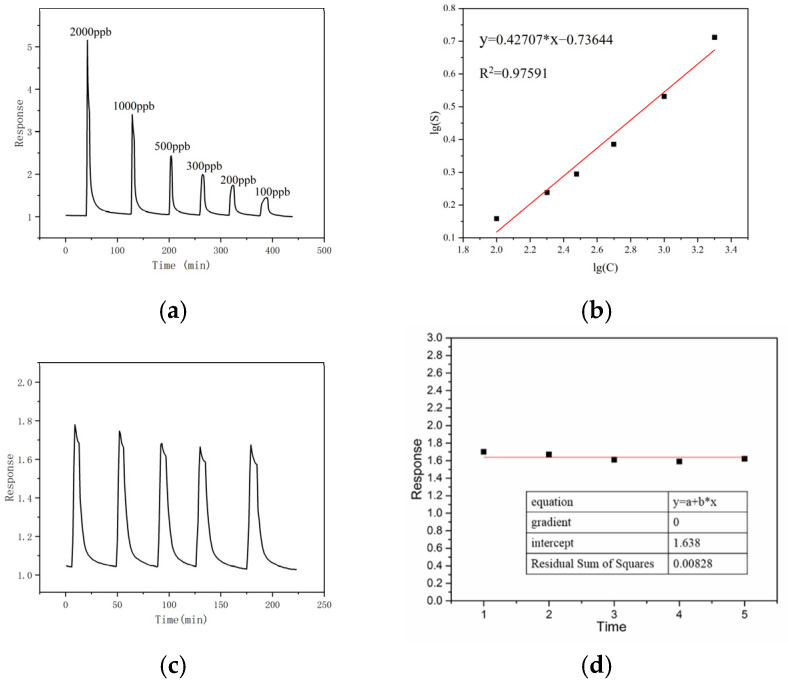
(**a**,**b**) The responses of sample 2 (1:8 In_2_O_3_:SnO_2_) to ethanol and (**c**,**d**) the repeatability of this sample’s response to 200 ppb ethanol.

**Figure 9:**
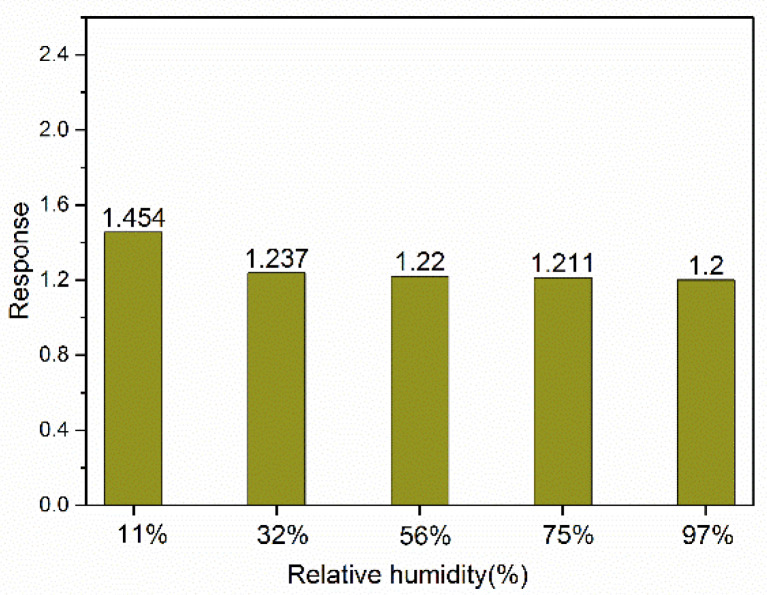
Baseline resistance and balancing resistance to 200 ppb ethanol of In_2_O_3_/SnO_2_ sensor 2# under different relative humidities.

The previous tests showed that sample 2# performed best. As shown in Figure 8, sensor 2# had an obvious response to a low concentration of ethanol and had good repeatability. The response of the In_2_O_3_/SnO_2_ sensor to 100 ppb ethanol was 1.41, while the response of the pure In_2_O_3_ sensor to 100 ppb ethanol was 1.21. The baseline resistance and the resistances of In_2_O_3_/SnO_2_ sensor 2# to 200 ppb ethanol at different relative humidities at 150 °C are shown in Figure 9. The sensors can also work in a humid environment.

### 3.3. Discussion

First of all, we analyzed the process and yield of the hydrothermal preparation. About 0.64 g of composite material can be prepared each time using a 100 mL reactor, and the total preparation time is about 48 h. If a larger reactor were used, a higher yield could be obtained per preparation. In addition, there are several times during the preparation process that require natural cooling. It can be assumed that using a heat dissipation device would shorten the preparation time.

Next, we will present a detailed discussion and analysis based on the gas test results. The ethanol gas sensor based on the In_2_O_3_/SnO_2_ nanocomposite prepared by hydrothermal synthesis demonstrated high responses, a low detection limit, and a low operating temperature.

As shown in Table 2, the ethanol sensor based on In_2_O_3_/SnO_2_ in this work has several advantages. In_2_O_3_/SnO_2_ has a lower working temperature, consumes less power, and can detect ethanol at a lower concentration of 100 ppb. Compared to pure In_2_O_3_, In_2_O_3_/SnO_2_ has a higher response to 2 ppm ethanol. This In_2_O_3_/SnO_2_-based sensor has good repeatability and can operate in humid environments.

According to the detection concentration and the signal-to-noise ratio measured at 100 ppb, the theoretical detection limit was calculated. The LOD (limit of detection) of the sensor is approximately 1.5 ppb.

**Figure 10:**
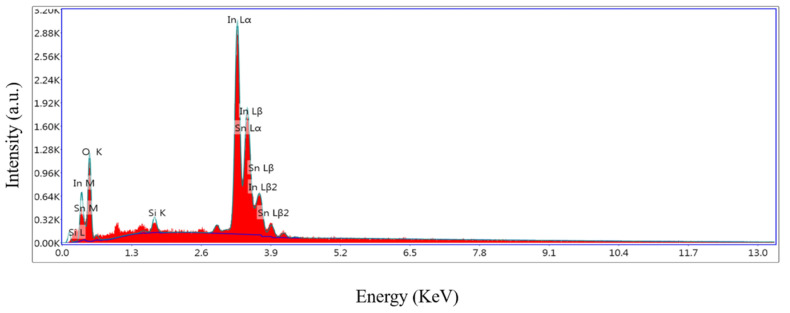
EDS of sample 2# In_2_O_3_/SnO_2_ nanocomposite.

**Figure 11:**
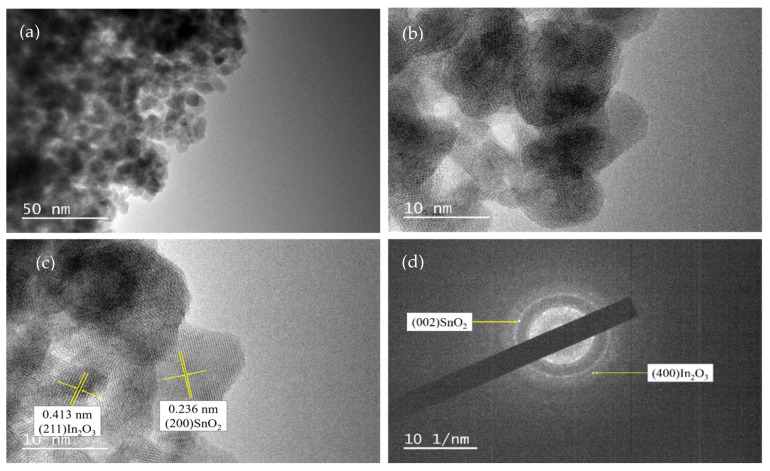
(**a**,**b**) HRTEM images of In_2_O_3_/SnO_2_ (sample 2#); (**c**) d-spacing; and (**d**) SAED patterns.

Figure 10 shows the distribution of the O, Sn, and In elements in the sample 2# In_2_O_3_/SnO_2_ nanocomposite based on EDS (energy-dispersive spectroscopy). Figure 11 shows the sample 2# In_2_O_3_/SnO_2_ nanocomposite as characterized by a JEOL JEM-2100 TEM (transmission electron microscope). As shown in Figure 11, at a higher magnification, the nanocomposite can be clearly seen as a particle with a diameter of about 10 nm. The two metal oxides are evenly distributed in the picture. As shown in Figure 11c, a d-spacing of 0.236 nm was attributed to the SnO_2_ (200) plane and 0.413 nm was assigned to the (211) plane of In_2_O_3_. Figure 11d shows the SAED (selected-area electron diffraction) patterns of In_2_O_3_/SnO_2_.

These results agree well with the structural results obtained from the XRD patterns. Next, we discuss the mechanism of the sensitive material.

When the nanomaterial is in air, the surface of the material adsorbs oxygen in the air, forming oxygen vacancies, which leads to an increase in the material resistance. When the material comes into contact with ethanol gas, ethanol reacts with oxygen to produce gains and losses of electrons, forming a large number of free electrons, which causes the resistance of the material to decrease, and the concentration of ethanol can be obtained by testing the resistance change of the material. When the temperature is above 150 °C, oxygen mostly exists as $O^{-}$. The specific reaction process is depicted in Equations (2)–(6).

(2) $$O_{2\left(gas\right)}\to O_{2\left(adsorbed\right)}$$

(3) $$O_{2(adsorbed)}+e^{-}\to O_{2(adsorbed)}^{-}$$

(4) $$O_{2(adsorbed)}^{-}+e^{-}\to 2O_{\left(adsorbed\right)}^{-}$$

(5) $$O_{\left(adsorbed\right)}^{-}+e^{-}\to O_{\left(adsorbed\right)}^{2-}$$

(6) $$C_{2}H_{6}O_{\left(gas\right)}+6O^{-}\to {3H}_{2}O_{\left(gas\right)}+2CO_{2(gas)}+6e^{-}$$

The composite material of In_2_O_3_/SnO_2_ has a larger specific surface area, which enables the sensor to absorb more gas molecules when contacting ethanol, acquiring electrons more quickly and reducing the resistance. In addition, the work function and band gap of SnO_2_ and In_2_O_3_ are W_1_ =4.7 eV and Eg_1_ = 3.5 eV, and W_2_ = 4.3 eV and Eg_2_ = 3.6 eV, respectively [46].

**Figure 12:**
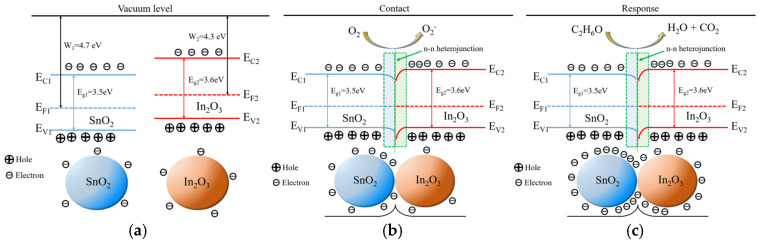
Schematic diagrams and energy band structures of the In_2_O_3_/SnO_2_ nanocomposite: (**a**) before contact; (**b**) in air; and (**c**) in ethanol.

As shown in Figure 12, the band gaps of the two metal oxides are different, leading to band bending at the junction of the two materials. As a result, the resistance of the material increases, and the test results confirmed this. Compared with pure In_2_O_3_, the two materials In_2_O_3_ and SnO_2_ can form an n-n heterojunction through hydrothermal synthesis, so when exposed to ethanol gas, the sensor can form an electron depletion zone, releasing more free electrons, thereby improving the gas-sensitive performance of the material as a whole.

## 4. Conclusions

An ethanol sensor based on In_2_O_3_/SnO_2_ was prepared by hydrothermal synthesis. Then, characterization of the material and tests of the gas-sensitive properties of the sensor were carried out. The experimental results showed that the sensor has a low detection limit of 100 ppb and a higher response to ethanol compared to pure In_2_O_3_. The In_2_O_3_/SnO_2_-based sensor also has good repeatability and can work in a humid environment.

Due to the advantages above, this ethanol sensor has a potential application in exhaled breath analysis. In the future, we will continue to investigate the responses of this nanocomposite-based sensor and will work to improve the accuracy of detecting trace ethanol in the complex VOCs in exhaled breath.

## Figures and Tables

**Table 1:** Mark numbers of different materials.

Elements’ Mol Ratios	Sn	100%	80%	60%	40%	20%	0
	In	0	20%	40%	60%	80%	100%
Sample numbers		1#	2#	3#	4#	5#	6#

**Table 2:** Comparison of gas-sensing characteristics of ethanol sensors.

Materials	Target Gas	Concentration	Response	Temperature	Ref.
In_2_O_3_	Ethanol	2 ppm	3.4	300 °C	[35]
ZnO-In_2_O_3_	Ethanol	50 ppm	170	240 °C	[36]
SnO_2_	Ethanol	50 ppm	16	200 °C	[38]
CuO	Ethanol	100 ppm	1.76	250 °C	[43]
Mo-SnO_2_	Ethanol	100 ppm	46.8	220 °C	[44]
SnO_2_-CuO	Ethanol	100 ppm	8	320 °C	[45]
In_2_O_3_/SnO_2_	Ethanol	100 ppb 2 ppm	1.4 5	150 °C	This work

## Data Availability

Data will be made available on request.
